# What is known about palliative care in adult patients with allogeneic stem cell transplantation (allo-SCT)?

**DOI:** 10.1007/s00277-021-04538-4

**Published:** 2021-05-06

**Authors:** Steffen T. Simon, Anne Pralong, Michael Hallek, Christoph Scheid, Udo Holtick, Marco Herling

**Affiliations:** 1 Faculty of Medicine and Cologne University Hospital, Department of Palliative Medicine, University of Cologne, 50924 Cologne, Germany; 2 Faculty of Medicine and Cologne University Hospital, Center for Integrated Oncology Aachen-Bonn-Cologne-Duesseldorf (CIO ABCD), University of Cologne, Cologne, Germany; 3 Faculty of Medicine and Cologne University Hospital, Center for Health Services Research (ZVFK), University of Cologne, Cologne, Germany; 4 Faculty of Medicine and Cologne University Hospital, Department of Internal Medicine I, University of Cologne, Cologne, Germany; 5 Clinic of Hematology and Cellular Therapy, University of Leipzig, Leipzig, Germany

**Keywords:** Stem cell transplantation, Allogeneic transplantation, Quality of life, Palliative medicine, Supportive care

## Abstract

Patients undergoing allogeneic stem cell transplantation (allo-SCT) are given a real chance of cure, but at the same time are confronted with a considerable risk of mortality and of severe long-term impediments. This narrative, non-systematic literature review aims to describe the supportive and palliative care needs of allo-SCT recipients, including long-term survivors or those relapsing or dying after transplantation. It also evaluates the feasibility and effectivity of integrating palliative care early in transplant procedures. In this appraisal of available literature, the main findings relate to symptoms like fatigue and psychological distress, which appear to be very common in the whole allo-SCT trajectory and might even persist many years post-transplantation. Chronic GvHD has a major negative impact on quality of life. Overall, there is a paucity of research on further issues in the context of allo-SCT, like the distress related to the frequently unpredictable post-transplant trajectory and prognosis, as well as the end-of-life phase. First randomized controlled results support the effectiveness of early integration of specialized palliative care expertise into transplant algorithms. Barriers to this implementation are discussed.

## Introduction

The long-term survival after allogeneic hematopoietic stem cell transplantation (allo-SCT) has been increasing, which is mostly attributable to continuous advancements in procedural aspects and supportive measures [1–3]. Despite these improvements, the overall mortality associated with allo-SCT remains high. The 5-year survival after allo-SCT, all diagnoses included, is about 50% [4]. Complications of the allo-SCT often lead to severe symptomatology, requiring optimal supportive and palliative management.

Palliative care aims to improve “the quality of life of patients and their families facing the problem associated with life-threatening illness, through the prevention and relief of suffering by means of early identification and impeccable assessment and treatment of pain and other problems, physical, psychosocial and spiritual” [5]. Relieving symptoms and managing other causes of suffering in cancer patients is mainly the task of oncologists and primary care teams in the sense of primary palliative care, but in complex situations, involvement of specific palliative care expertise will be necessary [6–8]. The early integration of such specialist palliative care into concepts of cancer therapy has become integral part of standard cancer care as recommended in several oncology and palliative care guidelines [6, 8–11]. Mounting evidence attests the effectiveness of early specialist palliative care in patients with solid tumors in the control of symptom intensity and with positive impacts on quality of life [6, 8, 9, 12, 13].

In contrast to solid cancers, specialist palliative care services are less frequently used in patients with hematological malignancies [14]. These patients receive more invasive therapies, even until shortly before death and are less likely to be informed about near death [14–16]. These facts from clinical practice are reflected in the current state of research in this area. There are few prospective studies that investigated the effect of early-integrated palliative care into the therapeutic algorithms in hematology [17, 18], in particular in allo-SCT [19], although recent results from randomized controlled trials indicate growing interest and research activity in that field [20–22].

Particularly recipients and survivors of an allo-SCT deserve special attention, because of the high morbidity and mortality associated with this modality. There is a high need to best identify and manage the many unmet needs in the field of supportive and palliative care during the whole transplant trajectory. In this non-systematic review, we aim to provide an overview on the current literature and state of the art on supportive and palliative care in patients with allo-SCT, which includes (1) symptoms, psychosocial distress, quality of life, burden related to the unpredictable illness trajectory and prognosis, issues in the last phase of life, support of family caregivers and (2) the integration of specialist palliative care services into transplant algorithms. For this purpose, we conducted a non-systematic search in the database MEDLINE (via PubMed), combining search terms on allo-SCT and on palliative care, psycho-oncology, uncertainty, fear of recurrence, or death anxiety. Further publications were identified by cross-referencing from publications listed in relevant studies and by citation tracking via the PubMed link for “related articles.” We included (systematic) reviews as well as original research articles (presented in Table 1). We considered studies including adult patients having undergone allo-SCT and/or their informal caregivers. Reviews or primary studies on hematology or SCT in general were also included if they reported specific results on allo-SCT. Primary studies with more than two-thirds of allo-SCT participants were considered as well. In this review, we also highlight relevant research gaps.

**Table 1:** Primary studies identified by the PubMed search on palliative and supportive care needs in allo-SCT recipients and on specialist palliative care integration

First author, year, country	Study design	Patient population	Main outcomes	Main results on allo-SCT
Amonoo 2019 [23], USA	Sequential qualitative interviews	25 allo-SCT recipients at baseline and 21/25 at follow-up (100 days after SCT)	Positive psychosocial experience	Family support and deliberate participation in pleasant and meaningful activities were the two primary sources of positive psychological experiences after allo-SCT. At baseline, participants consistently reported gratitude for their donors while follow-up was consistently characterized by hope for cure. Participants related bidirectional relationships between positive psychological well-being experiences and completion of health behaviors over time.
Andersson 2011 [24], Sweden	Prospective longitudinal, comparative	202 SCT recipients: • Autologous: *n* = 145 (71%) • MAC: *n* = 25 (12%) • RIC: *n* = 32 (15%)	Symptoms and HRQL in the first year post-SCT	Overall: similar recovering in RIC and autologous groups; sign. worse scores in MAC group during the whole study period. At 1 year: symptom and functioning scores back to BL or better in RIC and autologous group; worse in the MAC group in 10 of the 29 scales.
Bergkvist 2016 [25], Sweden	Qualitative interviews	14 family caregivers of patients after allo-SCT	Family members’ life situation and experiences of care	The findings show the family members’ voice of the uncertainty in different ways, related with the unknown prognosis of the HSCT, presented as Being me being us in an uncertain time. Positive experiences such as freedom and security from home care were identified. Different strategies such as adjusting, having hope, and live in the present used to balance to live in an uncertain time.
Bergkvist 2020 [26], Sweden	Qualitative interviews	14 family caregivers of patients 16 weeks after allo-SCT	Family caregivers’ experiences of providing and receiving support during allo-HSCT	Four prerequisites for family caregivers’ ability to provide support: Individual characteristics influence the ability to be supportive, social context influences the ability to be supportive, medical information provides knowledge and a sense of participation and interaction with the healthcare organization provides a sense of participation. Family caregivers’ risk of experiencing a stronger sense of uncertainty and lack of participation is higher in the absence of the above-mentioned prerequisites.
Bevans 2008 [27], USA	Prospective longitudinal	76 allo-SCT recipients (54% RIC)	Symptoms in the first 100 days post-allo-SCT	Overall: multiple symptoms and high symptom distress at days 0 and 30 after transplant conditioning. By day 100: number of symptoms and total symptom distress comparable to BL. Prevalent symptoms: • BL: fatigue (68%); worry (68%) • Day 0: appetite change (88%); fatigue and insomnia (86%); highest number of symptoms (*Mdn* = 8 (1–11)] • Day 30: fatigue (90%) • Day 100: fatigue (81%) Symptom distress: highest at day 0, followed by day 30
Bevans 2014 [28], USA	Prospective longitudinal	171 allo-SCT survivors ≥ 3 years	Physical and mental health status, HRQL and physical symptom distress, ≥ 3 years after allo-SCT	Overall: mean scores for physical and mental health and HRQL preserved relative to population norms at 3 years or more. Physical symptom distress: sign. predictor of worse physical and mental health status. Distress was significantly lower than healthy population values, and clinically meaningful: 73 (42.7%) subjects reported high levels of symptoms distress (>15), with a mean number of prevalent symptoms ranging across time from 12 (SD ± 3) to 14 (SD ± 4) symptoms (mean number of symptoms across the cohort: 8 to 10)
Busemann 2017 [29], Germany	Retrospective chart review	123 patients who died after allo-SCT	Somatic, psychic, and Spiritual needs	• About 50% of patients did not live more than 5 months. Two-thirds died within 14 months after SCT. • Major symptoms: weakness (48% at last admission; 40% 7 days before death), fatigue, and need for aid at daily activities. Severe pain, dyspnea, and obstipation were rare. • Measures of intensive care and i.v.-drug administration were applied to a significant proportion of patients. • Switch to a PC concept in 75/123 (61%) cases; 35% of artificial ventilation in the final phase of life.
Button 2014 [30], Australia	Retrospective chart review; survey among advanced practice nurses	40 allo-SCT recipients who relapsed and died	Patients characteristics, characteristics of PC/end-of-life care	• 50% of patients seen by PC service • Survey participants felt end-of-life discussions were left until the terminal phase. Participants believed early PC integration was beneficial for patients and their family.
Cappell 2018 [31], USA	Retrospective chart review	422 patients who died after allo-SCT	Documentation and timing of advance directive (AD); use of ICU and of mechanical ventilation after SCT; location of death	• Prevalence: AD documentation prior to death: 44%. • Associations: patients with ADs less likely to use the ICU during the transplant course (41% for patients with Ads versus 52% of patients without Ads; *p* = .03); less likely to receive mechanical ventilation at any point after SCT (21% versus 37%, *p* < .001); decreased ICU use at the end-of-life; more likely to die at home or in hospital as opposed to in the ICU (odds ratio, .44; 95% CI .27/.72).
Cohen 2012 [32], USA	Prospective longitudinal, comparative	164 SCT recipients: • Autologous: *n* = 49 (38%) • MAC: *n* = 49 (30%) • RIC: *n* = 53 (32%)	Symptoms and QoL, in the first 100 days post-SCT	Overall: MAC group showed more severe sleep disturbance and poorer QOL than autologous group. Patients with acute GvHD had sign. more severe symptoms. At day +100 post-allo-SCT: • RIC group: higher score for the “worst five symptoms” (fatigue, sleep disturbance, physically weak, drowsiness, and lack of appetite) and for physical weakness than autologous group • MAC group: higher scores for the “worst five symptoms,” for fatigue, and for physical weakness than autologous group
Cusatis 2020 [33], USA	Prospective longitudinal	184 allo-SCT recipients and survivors until 1-year post-allo-SCT	Decisional regret about allo-SCT and association with QoL and clinical outcomes (relapse, GvHD), before allo-SCT, at day +100, at month +6 and +12 post-allo-SCT	Decisional regret: 6% to 8% of patients expressed regret at day +100, month +6 and +12 post-allo-SCT; a total of 15% expressed regret at any time point. Associations: Regret was associated with: • Lower QoL scores at 6 months and 12 months (*p* < .001) • Lower baseline QoL and social well-being • Disease recurrence: 17.5% (95% CI, 5.5–29.7%) greater risk
Dunn 2016 [34], UK	Qualitative interviews	16 patients after allo-SCT	Live experience after allo-SCT	The Immediacy of Illness and Existential Crisis developed from participants’ experiences of critical events accompanied by enduring uncertainty continuing into the recovery period. Participants suffer major disruption to their lives physically, psychosocially, and emotionally, including facing their own mortality, without a sense of when they may resume the normality of their former lives.
El-Jawahri 2015 [35, 36], USA	Prospective longitudinal	90 SCT recipients and their family caregivers (FC): • Autologous: *n* = 30 • MAC: *n* = 30 • RIC: *n* = 30	QoL and mood of patients and FC during hospitalization for SCT (from day-6 pre-SCT to day+8 post-SCT) [36]; Prognostic understanding and its association with QoL and mood [35]	QoL: sign. decline of patients’ and FC QoL Mood: sign. increase of patients’ and FC depression; anxiety in patients and FC stable. Prognostic understanding: • 88.9% of patients and 87.1% of FC reported it is “extremely” or “very” important to know about prognosis. • Prognostic understanding: more optimistic than the oncologist’s in 77.6% of patients and 71.7% of FC (*p* < 0.0001) • Association with QoL/mood: patients with a concordant prognostic understanding with their oncologists had worse QoL (*β* = − 9.4, *p* = 0.01) and greater depression at BL (*β* = 1.7, *p* = 0.02) and over time (*β* = 1.2, *p* < 0.0001).
El-Jahwari 2016 [20] / 2017 [21], USA	Randomized controlled trial, non-blinded	160 SCT recipients • Intervention: inpatient PC integrated with SCT care: *n* = 80 • Control: standard SCT care: *n* = 80 stratified by transplant type: autologous 50%, MAC 19%, RIC 31%	Symptoms (fatigue, anxiety, depression), symptom burden and QoL, at 2 weeks post-hospitalization [20], and 6 months [21] post-SCT	PC (intervention) group: • Sign. improvement in QOL (Functional Assessment of Cancer Therapy - Bone Marrow Transplantation, FACT-BMT: mean difference between groups − 6.82 (95% CI: − 13.48 to 0.16; *p* = 0.045), symptom burden, depression and anxiety symptoms at 2 weeks post-SCT • Sign. improvement in depression symptoms and post-traumatic stress symptoms at 6 months after SCT • Caregivers: improvement in depression symptoms at 2 weeks The effect of the intervention did not differ by transplant type.
Esser 2017 [37–39] / Kuba 2017 [40, 41] / Sarkar 2014 [42], Germany	Prospective longitudinal, comparative	Allo-SCT recipients/ survivors: • T0 (pre-condition-ing) : *n* = 239 • T1 (day 100 post-SCT): *n* = 150 • T2 (1 year): *n* = 102 • T3 (5 years): *n* = 45 Control group (general population) drawn from large representative samples in Germany, age- and gender-matched	Symptoms (fatigue [37], anxiety and depression [40], others [38]), PTSD [39], cancer-and-treatment-specific distress [41], and fear of recurrence [42]	Global fatigue: • T0 to T1: sign. increase (*t* = 3.85, *p* < 0.001) • T1 to T2: sign. decrease (*t* = − 2. 92, *p* = 0.004), then stable • T0 to T3: non-sign. difference (*t* = 0.68, *p* = 0.497). • Allo-SCT vs. general population: higher global fatigue at T0 (*t* = − 6.02, *p* < 0.001) and T3 (*t* = − 2.50, *p* = 0.014); meaningful effect sizes (*d* ≥ 0.5). • Predictors of fatigue: acute and chronic GvHD at T1 and T2, respectively Depression: • T2 to T3: sign. increase of prevalence rates from 12 to 30% • Allo-SCT vs. general population: sign. lower RR at T0 (RR = 0.56; 95% CI 0.4/0.8) and T2 (RR = 0.47; 95% CI 0.3/0.8); at T1 and T3: non-sign. Anxiety: • T0 to T1: sign. decrease of prevalence rates of anxiety from 29 to 19% • T1 to T2/T3: stable • Allo-SCT vs. general population: sign. increased at T0 (RR = 1.31; 95% CI 1.02/1.68); from T1 on: non-sign. Symptom clusters (SC): 3 stable SC (present at 3 consecutive time points): rest-tired-weak-dyspnea-loss of appetite, tense-worried-irritable-depressed, and nausea-vomiting PTSD symptomatology: • 15% met the criteria for PTSD at least once during the course of assessment. • 52% showed diagnostic relevant levels of intrusion, 30% of avoidance, and 33% of arousal at least once. • Sign. predictors at all time points: being impaired by pain (*γ* = 2.89, *p* < 0.01), pain level (*γ* = 0.63, *p* = 0.02), and being female (*γ* = 3.81, *p* < 0.01) • Sign. predictors at T2: acute and chronic GvHD, longer hospital stay Cancer-and-treatment-specific distress (CTXD): sum score of CTXD was highest at T0, then decreased by T1 (*γ* = −.18, 95% CI −.26/−.09), and by T2 (*γ* = −.10, 95% CI −.20/−.00). Subscales: uncertainty, family strain, and health burden were rated most distressing during SCT. Fear of recurrence: • Prevalence of high fear of recurrence: T0: 36%; T1: 24% of patients; T2: 23% • Predictors: T0: being married (*b* = 2.76, *p* = 0.026), female gender (*b* = 4.45, *p* < 0.001), and depression (*b* = 4.44, *p* < 0.001) were significantly associated with FCR at baseline. T1: depression (*b* = 6.46, *p* < 0.001). T2: female gender (*b* = 6.61, *p* = 0.008), higher depression (*b* = 4.88, *p* = 0.004) • Associations: fear of recurrence and lower physical functioning (*p* = 0.019), role functioning (*p* = 0.003), emotional functioning (*p*=0.001), cognitive functioning (*p* = 0.003), social functioning (*p*=0.001), and global QoL (*p* < 0.001).
Grulke 2010 [43], Germany	Prospective longitudinal; survey among physicians	136 allo-SCT recipients (72 died during the 2-years follow-up)	Comparison of physician prognostic estimates and patient survival	Physicians’ estimates associated with overall survival (univariate Cox regression: hazard ratio = 1.51, 95% CI = 1.24–1.82)
Han 2019 [44], USA	Retrospective chart review	21 458 hospitalized SCT recipients (40% allo-SCT)	General prevalence, temporal trends, and predictors of PC use from 2008 to 2014	Rate of PC use: • Among SCT: 1.30% (278/21 458) • Among allo-SCT: 1.95% Rate of PC use from 2008 to 2014: • Among SCT: sign. increase (annual percentage change: 12.96%) • Among allo-SCT: sign. increase (annual percentage change: 16.45%) Predictors of higher PC use in allo-SCT: higher comorbidities (OR = 3.19; 95% CI 1.77/5.77; *p* = .0001) and GvHD (OR = 2.04; 95% CI 1.36/3.06; *p* = .0006)
Hefner 2014 [45], Germany	Cross-sectional	41 allo-SCT recipients /survivors (mean time after transplantation: 614 days (25 to 2 070 days))	Distress (anxiety, fear of progression, depression, PTSD)	Prevalence rates: symptoms of distress in total: *n* = 18 (44%), of which: symptoms of anxiety: *n* = 11 (27%); fear of progression: *n* = 12 (29%); symptoms of depression: *n* = 11 (27%); symptoms of PTSD: *n* = 6 (15%) Associations: age < 55 years significantly associated with fear of progression (*p* = 0.004). No association between distress and acute or chronic GvHD or time after allo-SCT
Heinonen 2005 [46], Finland	Survey	109 allo-SCT recipients	Identification of stressors	Identification of 8 stress clusters (from the most severe to the least severe): change of life and impact of long-lasting treatment; side effects; distress related to treatment outcome and physiological status; family-related stress; fear of death and depressive thoughts; other concerns; negative social support; and stress related to lack of information and the medical staff.
Loggers 2016 [47], USA	Prospective longitudinal	22 allo-SCT recipients	Feasibility and acceptability of pre-SCT early PC	• Comfort with early PC: high (82% very comfortable). • Mood, sense of hope: stable or improved subjective mood and sense of hope • Follow-up surveys (60 day 60 and 90): 4 (20%) admitted to the ICU before day 100 and 3 (15%) received life-support measures. 5 (25%) died with median follow-up of 14 months.
Scherwath 2013 [48], Germany	Prospective longitudinal	102 allo-SCT recipients	Cognitive function (attention, memory, executive function, fine motor function), at T0, T1 (100 days), and T2 (1 year)	Comparison with test norms: below test norms in up to 50% of the test scores. Patients were mostly impaired on word fluency (24%, T0), fine motor function, and verbal delayed recall (19% each, T2). Over time: partial improvement in performance (i.e., visual span forward, verbal learning, and word fluency). However, from T0 to T2, 16% of the patients showed reliable decline on ≥3/14 test scores. Associations: no associations with conditioning intensity, total body irradiation, GvHD, cyclosporine treatment, and length of hospital stay for most of neuropsychological subtests
Schulz-Kindermann 2007 [49], Germany	Prospective longitudinal	39 allo-SCT recipients	Cognitive function (attention, memory, executive function), at T0 and T1 (100 days)	Comparison with test norms: mostly no sign. differences at T0 or T1, except verbal long-term memory (T0 and T1) and visual working memory (T1) below norm. Over time: sign. prolonged simple reaction time Associations: no association with extent of pretreatment, GvHD, or conditioning protocol
Valkova 2016 [50], Czech Republic	Retrospective chart review	590 allo-SCT recipients (64% MAC, 36% RIC)	QoL (median time from allo-SCT to questionnaire completing: 3.8 years [range: 0.2 to 21.6])	Overall QoL: lowest score immediately after allo-SCT, with subsequent higher score after 100 days, followed by lower score in the period between 1 and 2 years, and then a sustained increase. Scores: 73% of the maximum values at 1 year, 80% at 3 years, and 82% at 5 years. Predictors of worse QoL: acute and chronic GVHD in the last 6 months (regardless of the extent); increasing age
Wang 2017 [51], USA	Retrospective chart review	602 SCT recipients (39% allo-SCT)	Advanced directives (AD) and/or Physician Orders for Life-Sustaining Treatments (POLST) completion, PC consultation, hospice enrollment	Prevalence of PC consultation: 19% (*n* = 114) of SCT patients, with 83% (*n* = 95) occurring in the hospital. Sign. difference between transplant types with 11% of allo-SCT (*n* = 68) and 8% of autologous SCT (*n* = 46) receiving PC AD/POLST completion rate: • 44% of SCT patients (*n* = 267) • Allo-SCT: sign. greater rate than autologous SCT (OR, 1.56; 95% CI, 1.12 to 2.17; *p* = .008) Hospice enrollment rate: 15% (*n* = 17)

*Abbreviations*: *allo-SCT*, allogeneic stem cell transplantation; *BL*, baseline; *CI*, confidence interval; *FC*, family caregivers; *GvHD*, graft-versus-host disease; *HRQL*, health-related quality of life; *ICU*, intensive care unit; *MAC*, myeloablative conditioning; *OR*, odd ratio; *PC*, palliative care; *PTSD*, post-traumatic stress disorder; *QoL*, quality of life; *RIC*, reduced-intensity conditioning; *RR*, relative risk; *SDS*, symptom distress score; *sign*., significant

## Symptoms, psychosocial distress, and quality of life

The impact of allo-SCT on symptoms and quality of life has been the topic of numerous reviews [19, 52–64] and more recent primary studies (see Table 1). The most prevalent physical symptoms in the first 3 months after allo-SCT are fatigue, sleep disturbances, nausea, lack of appetite, and indigestion (obstipation or diarrhea) [27, 32, 56]. Their incidences peak during the period from transplantation until 1 month thereafter [27, 32]. At about 1 month, physical symptoms start to decrease and physical functions noticeably improve [24, 32, 59] and tend to reach baseline levels at about 1 year post-transplantation [24, 59]. Overall, allo-SCT recipients after a myeloablative conditioning seem to show more symptoms and a slower recovery than those with reduced-intensity conditioning (RIC) [24, 32, 57].

Symptoms, which persist at least 1 year after allo-SCT compared with population norms and non-cancer comparison groups, include fatigue, sleeping disorders, pain, and sexual dysfunction [28, 53, 56], with fatigue being associated with female gender and younger age, as well as acute and chronic GvHD [37, 54]. In a prospective multicenter study with 239 patients, fatigue was identified as the most persistent and severe symptom even 5 years after allo-SCT; it was also the most important predictor of a reduced quality of life [38]. Even if global fatigue returned to pre-transplantation levels after about 1 year and remained stable for the next 5 years, it was still significantly more prevalent than in the general population [37].

Patients with acute graft-versus-host disease (GvHD) have a higher burden of physical symptoms and inferior physical well-being [32, 50]. Chronic GvHD is an important issue at long term and predicts very strongly the persistence of impaired physical functions [28, 54, 57].

Psychological distress is among the most prevalent symptoms in patients with allo-SCT [52–54]. Anxiety has been described as frequently increased especially before transplantation [40], while depression becomes prominent post-transplantation, with an increased prevalence until 10 years post-allo-SCT [40, 54]. Post-traumatic stress disorders (PTSD) is a major concern in about 5 to 19% of SCT survivors, independent of the kind of transplantation [21, 39, 54, 65]. Cognitive disorders are relatively common among patients that undergo and underwent an allo-SCT, and are frequent even before the transplantation [48, 49]. A cognitive decline from pre- to post-(allo-)SCT could not be demonstrated in a meta-analysis of 404 patients [58]. Compared to autologous SCT recipients, patients with allo-SCT appear to have a higher risk for long-term psychological distress, especially those with chronic GvHD [52, 54]. Acute and chronic GvHD both have a negative impact on mental health [50, 59].

The quality of life after transplantation has been reported to return to pre-allo-SCT levels after about 1 year [50, 54, 59]. Risk factors for poorer quality of life include young age, female gender, low educational level, limited social support, physical symptoms, and depression as well as acute and chronic GvHD [54]. Chronic GvHD is the major cause of impaired quality of life in allo-SCT survivors [55]. Patients with poorer quality of life have a higher risk of expressing regrets about their decision to undergo the allo-SCT procedure [33].

The effectiveness of early integration of specialist palliative care on symptom burden, psychological distress, and quality of life was examined in a randomized controlled trial (RCT) with 160 SCT recipients— to our knowledge the only one to date in the context of SCT [20, 21]. The interventional group received a consultation by a palliative care consultant at least twice a week during the time of hospitalization and the control group received standard transplant care. Randomization was stratified by transplant type (autologous, myeloablative allogeneic, or RIC allogeneic) with half of the patients receiving an allo-SCT. Transplant type was not found to be a mediator of the effect of the intervention. In the interventional arm, patients showed less reduction in quality of life at 2 weeks after admission, which was significant and clinically relevant (Functional Assessment of Cancer Therapy - Bone Marrow Transplantation [FACT-BMT]: mean difference between the groups −6.82 [95% CI: −13.48 to 0.16; *p* = 0.045]). Among secondary endpoints, at 2 weeks post-SCT, anxiety was significantly lower in the interventional group, while depression and symptom burden increased significantly less, and levels of fatigue did not differ. At 3 months post-SCT, quality of life was higher and symptoms of depression were lower in the interventional group [20]. At 6 months, the significant differences for the parameter of depression persisted and symptoms of a post-traumatic stress disorder were lower in the interventional arm [21].

## Family caregivers

The impact of allo-SCT also extends to the family caregivers, who play a key role in caring for patients after discharge from the hospital [52, 64]. Most of them are spouses. They experience an increased level of anxiety and symptoms of depression as well as physical symptoms like fatigue, sleep disturbances ,weight loss, and changes in marital and social life [64]. Their own burden might also have an impact on patients’ outcomes, like mental health or number of hospitalizations [64]. More research is needed to better understand the interrelation between the needs of patients and their caregivers, and to develop targeted interventions. First results of psychological interventions are promising [66, 67]. The early integration of specialized palliative care in standard allo-SCT procedures might also positively affect caregivers’ outcomes, as shown in the mentioned RCT [20]: caregivers of patients in the intervention group had a significantly smaller increase in depression at 2 weeks as compared to caregivers of patients in the standard care arm; however, no significant differences in quality of life or anxiety were found at that time point.

## Unpredictable illness trajectory and prognosis: uncertainty, fear of recurrence, and death anxiety

Allo-SCT has a high potential to provide long-term remissions of the underlying hematologic disease, but it is also associated with a high mortality [1, 2, 4]. In this context, uncertainty, death anxiety, and fear of recurrence are further sources of distress that patients experience in the context of an allo-SCT [41, 42, 46].

In allo-SCT recipients, uncertainty emerges as a main cause of burden and suffering [25, 26, 34–36, 43]. In a prospective cohort of 239 patients undergoing allo-SCT, uncertainty was found to be one of the predictive factors for symptoms of post-traumatic stress disorder before, 3, and 12 months after transplantation [41]. The authors hypothesized that the high uncertainty level may be due to the complex and unpredictable nature of allo-SCT, which is in line with findings on the first descriptions of patient’s uncertainty in unspecific illness situations [68–70]. Mishel, for instance, delineated four forms of uncertainty in a general context of illness: ambiguity regarding the state of the illness, complexity of treatment, lack of information, and unpredictability of prognosis [70]. Uncertainty in advanced illnesses like metastatic cancers has been addressed by various authors [68, 71, 72]. They confirm Mishel’s four forms of uncertainty, with complexity and unpredictability of illness being the most relevant factors at the end of life [68]. However, the exact components of uncertainty in the allo-SCT population and the ways to communicate and to manage still need to be explored [69, 73].

Fear of recurrence has been defined as the “fear, worry or concern relating to the possibility that cancer will come back or progress” [74]. Although it can be considered so far as a normal response to a cancer diagnosis, it may lead in some cases to dysfunctions like difficulties in sleeping and anxiety as well as to a decreased quality of life [75]. It therefore requires special consideration in clinical practice. The few research findings available for patients after allo-SCT indicate that about a third of them suffer from fear of recurrence [42, 45].

Fear of death is another important stress factor in allo-SCT [46]. However, it has hardly been explored to date and data from patients undergoing SCT are missing so far. A survey with 109 patients highlights fear of death as one of the main components of stress in allo-SCT, among other factors like change of life, side effects, or family concerns [46]. Prevalence rates for fear of death in patients with advanced cancer range from 30 to 80% [76, 77]. In patients treated for hematologic disease with a curative intent, preoccupation with death was found to be associated with higher anxiety and depression rates as well as with reduced coping [78]. Recently, interventions addressing death anxiety were developed (overview in [79]), but do not address allo-SCT recipients specifically.

To what extent fear of recurrence, death anxiety, and uncertainty are interrelated has been addressed rather rudimentarily [80–82]. Raising this issue could help to better understand distress in allo-SCT recipients and to optimize current management strategies.

## End-of-life care in patients with allo-SCT

When the underlying disease fails to respond to the allo-SCT modality or side effects such as infectious complications or refractory GvHD become incurable, the patient’s condition often deteriorates quickly. The goal of care then needs to be adapted to focus on an effective symptom control and to improve the quality of life. Life-prolonging treatments (including blood transfusion) are often stopped at this point. After this change of goals of care from life-prolonging to end-of-life care, the time until death is often short [29–31].

The last phase of life, including the dying phase, has been investigated in some studies on patients with hematologic cancers [16, 51, 83–86]. Their findings suggest a similar symptom burden and decline in the last 3 months of life as compared to patients with solid tumors [83, 85]. For end-of-life care in patients with allo-SCT, studies are missing so far, besides two retrospective chart reviews with participants who died after allo-SCT; the patients had an elevated burden of symptoms [29, 30]. Further research is necessary to explore distress and needs of patients with allo-SCT and their informal caregivers and to describe the illness trajectory in the last phase of life.

## Early integration of palliative care in the standard care of patients with allo-SCT

The early integration of specialist palliative care expertise in the transplant procedure aims at improving the experience of patients and caregivers throughout the individual trajectory, irrespective of whether that involves cure, chronic comorbidity, or death. This is achieved by improving symptom control, preventing and palliating psychosocial distress, enhancing patients’ and caregivers’ coping, and—if death nears—by caring through the last phase of life and by supporting caregivers’ bereavement [19–21, 87, 88]. This has been an emerging issue over the last years.

In general, the use of specialist palliative care services for patients with allo-SCT is rather infrequent, although it appears to have increased in recent years. A large retrospective US-American chart review of 21,458 patients hospitalized for SCT (40% with an allo-SCT) describes that 1.95% of allo-SCT recipients received specialized palliative care [44]. There was a significant increase in its use from 2008 to 2014 (annual percentage change 16.45%). Among the patients who died during hospitalization, the rate of involved palliative care services was only 33.40% [44].

Barriers resulting in the late and low rate of integration of specialist palliative care into the therapeutic algorithms in hematology have been described in general reviews on hematologic malignancies [14, 55, 89–91]. Their results are also applicable to the specific context of allo-SCT. They describe disease-related barriers like the unpredictable course of hematologic diseases or the unclear interface between a curative and palliative stage. Subjective factors play an important role as well, e.g., the perception that palliative care refers exclusively to the last phase of life, the fear to reduce patients’ hope, unrealistic expectations of patients, family members, and clinicians, or suboptimal collaborations between the disciplines of hematologists and palliative care experts [14, 55, 89–91].

Several models of integration of specialist palliative care in (allo)-SCT have been proposed, ranging from a selective palliative care consult ordered by the transplant team to the upfront integration of palliative care specialists in routine transplant care [19, 87]. However, such models of integration are implemented only in few institutions worldwide and thorough analyses of real-world clinical experiences are missing so far [19, 87]. Research on the efficacy of these models is also scarce. The RCT of El-Jawahri et al. mentioned previously confirmed the effectiveness of a consultative model on improving symptoms and quality of life [20, 21]. Further studies with a non-controlled prospective design supported the effectivity of other consultative models on palliative outcomes such as goals-of-care discussions, hospice referral, and keeping up hopes after talking to palliative care professionals [47, 92]. There is a need for research also on other models of integration such as co-rounding, embedded models with routine inclusion of palliative care, or models for allo-SCT survivors [19].

## Discussion

### Impact for clinical care

Integrating specialist palliative care early in the course of the allo-SCT procedure likely contributes to achieve the best possible symptom control, to help patients and caregivers to better cope with the illness and its prognosis (including distress by uncertainty, fear of recurrence, and death anxiety), and to optimize quality of life [17, 19, 47, 55, 87, 88]. Strategies for an early integration should be adapted to the specific issues of transplantation, as already implemented in some few projects [20, 21]. Structured interventional strategies (programs) along the whole transplant trajectory are likely to be more effective then sporadic on-demand involvements. A main issue around integrating palliative care in clinical SCT practice remains a regular contact and communication between transplant physicians and palliative care specialists in order to ensure a best possible collaboration between both disciplines. A further issue is the adequate information of patients about the advantage of integrating palliative care, even before transplantation.

### Impact for research

Overall, there is a high need for further research on burden, needs, and coping of allo-SCT patients and their caregivers, regarding physical symptoms as well as psychosocial distress when facing an uncertain prognosis and death. There is also an urgent need for interventional studies evaluating the most effective strategies to integrate specialist palliative care into the allo-SCT setting. It is furthermore highly warranted to systematically assess the effect of integration of psycho-oncology, psychosocial, and supportive care services in transplant procedures on outcome parameters such as quality of life in comparison to the impact of integration of specialized palliative care.

## Conclusion

During the past decades, survival of patients after allo-SCT has continually improved. Despite such better prognoses and the reduction of treatment complications, morbidity and mortality remain relatively high in this patient population. We need to better integrate palliative care into the standard care of patients with allo-SCT in order to improve symptom control, to enhance the prevention and support of psychosocial distress, and to provide best care at the end of life. Well-conducted studies around these topics are still scarce, although becoming more frequent, as far as we can judge from the results of this non-systematic review. They are urgently needed to provide best possible care and quality of life for patients undergoing an allo-SCT.
